# Multifunctional PVP/PEG Hydrogel Coatings Functionalized with Taxifolin for Surface Modification of Titanium-Based Substrates

**DOI:** 10.3390/ijms27135792

**Published:** 2026-06-26

**Authors:** Katarzyna Młyniec, Eliza Szymańska, Julia Sadlik, Edyta Kosińska, Katarzyna Haraźna, Krzysztof Miernik, Josef Jampilek, Agnieszka Sobczak-Kupiec

**Affiliations:** 1 Faculty of Materials Engineering and Physics, Department of Materials Engineering, Cracow University of Technology, 37 Jana Pawła II Av., 31-864 Krakow, Poland; 2 CUT Doctoral School, Faculty of Materials Engineering and Physics, Department of Materials Engineering, Cracow University of Technology, 37 Jana Pawła II Av., 31-864 Krakow, Poland; 3 Department of Analytical Chemistry, Faculty of Natural Sciences, Comenius University, Ilkovicova 6, 842 15 Bratislava, Slovakia; 4 Department of Chemical Biology, Faculty of Science, Palacky University, Slechtitelu 27, 779 00 Olomouc, Czech Republic

**Keywords:** composites, Ti6Al4V, taxifolin, hydrogel coatings

## Abstract

Surface functionalization of metallic implants is widely explored to enhance their performance and functionality. In this study, multifunctional hydrogel coatings based on poly(vinylpyrrolidone) and polyethylene glycol were developed and functionalized with a taxifolin (TAX) inclusion complex and collagen to introduce bioactive features. TAX, a naturally occurring flavonoid with antioxidant and anti-inflammatory properties, was incorporated using β-cyclodextrin to improve its stability and enable controlled release. The coatings were applied to titanium-hydroxyapatite composites and titanium sheet substrates to evaluate their applicability across surfaces with varying morphologies, ranging from porous to relatively smooth. The ceramic phase was modified with magnesium ions to enhance its bioactivity and better mimic the composition of natural bone tissue. FTIR and SEM analyses confirmed hydrogel formation and effective surface coverage. Degradation and incubation studies in simulated physiological environments demonstrated the material’s stability, while UV–Vis analysis indicated TAX release, highlighting the system’s potential as a carrier for flavonoid-based compounds. Indirect cytotoxicity studies using MC3T3-E1 preosteoblasts indicated low cytotoxicity and a favorable biological response of collagen- and taxifolin-modified systems. The developed coatings represent a versatile platform for surface modification of titanium-based biomaterials and demonstrate potential for application across substrates with diverse surface characteristics. Further studies are required to assess their biological potential.

## 1. Introduction

The continuous evolution of reconstructive surgery and orthopedics has established metallic biomaterials as the cornerstone of modern implantology [1,2]. Titanium and its premier alloy, Ti6Al4V, are widely used due to their unique combination of low density, high mechanical strength, and excellent corrosion resistance, which is attributed to the spontaneous formation of a stable, nanometric TiO_2_ passivation layer [3,4]. However, despite these beneficial properties, titanium implants are classified as relatively bioinert materials. Although titanium and the Ti6Al4V alloy generally support the osseointegration process, the limited bioactivity of their surfaces can, under unfavorable conditions, reduce the effectiveness of bone bonding and promote the formation of fibrous tissue [5,6,7]. Furthermore, the harsh environment of the human body, characterized by fluctuating pH and the presence of chloride ions, can induce tribocorrosion. This process leads to the systemic release of aluminum and vanadium ions, which are documented to cause long-term cytotoxicity, inflammatory reactions, and potential neurological complications [8,9].

To overcome these limitations, several strategies have been proposed. One of the most widely investigated approaches involves the incorporation of calcium phosphate-based ceramics, particularly hydroxyapatite (HAp), into metallic systems [10,11,12]. The impact of such modifications on the properties of metallic composites is multifaceted. From a mechanical standpoint, they can improve surface adhesion, osteoblast retention, and resistance to tribological wear [5]. From a physicochemical perspective, surface bioactivity increases, corrosion resistance improves, and the release of biologically harmful metal ions, such as Al or V, is reduced. In the biological realm, these modifications support bone formation, stimulate cell proliferation, inhibit bacterial growth, or accelerate tissue healing. Therefore, modern metallic composites are increasingly serving as carriers of bioactive components or sources of therapeutic ions that exert a regulatory effect on cellular processes [13]. One of the ions that has a significant impact, particularly in bone tissue regeneration, is magnesium. Mg^2+^ ions have a smaller ionic radius than Ca^2+^ ions [14,15,16]. This allows them to partially substitute within the HAp crystal lattice, leading to the formation of structural defects similar to those observed in natural bone. From a biological perspective, magnesium participates in the activation of numerous enzymes, influences calcium-phosphate metabolism, and regulates the function of osteoblasts and osteoclasts [17]. The presence of Mg^2+^ ions promotes the adhesion, proliferation, and differentiation of osteoblasts and increases the expression of osteogenic markers such as osteocalcin and alkaline phosphatase [18]. At the same time, it limits excessive osteoclast activity, supporting the maintenance of a balance between bone formation and resorption [19]. In implants, doping HAp with Mg^2+^ ions leads to a reduction in crystallinity and an increase in the material’s solubility, resulting in more dynamic ion exchange and better adaptation of the implant to bone remodeling processes [20]. For this reason, Mg^2+^ is a beneficial ion in the early stages of osteogenesis and in applications requiring high biocompatibility and a rapid biological response [21,22].

Another complementary approach to overcome these biological limitations is the development of bioactive surface coatings, particularly hydrogel-based systems [23,24]. Hydrogel coatings, due to their high water content and porous three-dimensional architecture, provide a biomimetic interface that closely resembles the natural extracellular matrix (ECM) [25,26]. In this study, a synergistic polymer blend of polyvinylpyrrolidone (PVP) and polyethylene glycol (PEG) was engineered. PVP is selected for its exceptional film-forming properties, high transparency, and strong adhesion to metallic substrates, while PEG acts as a plasticizing and stabilizing agent that inhibits non-specific protein adsorption (the “stealth” effect) [27,28,29,30,31]. The chemical cross-linking of these polymers via UV-induced photopolymerization allows for the creation of a tailored network that serves as a high-capacity reservoir for the controlled delivery of therapeutic agents directly to the surgical site [32,33,34].

A novel aspect of this research is the functionalization of the hydrogel matrix with Taxifolin (3,3′,4′,5,7-pentahydroxydihydroflavonol, TAX), a potent flavonoid derived from Siberian larch (*Larix sibirica*, Ledeb.) [35,36]. TAX is recognized for its superior antioxidant capacity, which neutralizes reactive oxygen species (ROS) generated during the inflammatory phase of post-operative healing [37,38]. Moreover, evidence suggests its potential in stimulating osteoblast activity and inhibiting osteoclastogenesis [39,40]. Additionally, TAX has antibacterial activity, which makes it an attractive component of implant coatings, especially in the context of preventing peri-implant infections [41,42,43]. The use of TAX as a coating modifier is consistent with the trend of using natural plant compounds as an alternative to synthetic pharmacological agents [40]. However, its therapeutic efficacy is traditionally hampered by poor aqueous solubility. To bypass this, we employed molecular encapsulation using β-cyclodextrin (βCD). The toroid-shaped CD molecule possesses a hydrophobic internal cavity and a hydrophilic exterior, forming an “inclusion complex” (TAX-βCD) [44]. This modification not only enhances the solubility of the flavonoid but also ensures a sustained, long-term release profile, preventing the “burst release” effect commonly seen in simpler systems [39,45]. To further bridge the mechanical-biological gap, the hydrogel system was reinforced with collagen (CLG), the most abundant structural protein in bone tissue. From an engineering perspective, CLG acts as a natural “fiber reinforcement” within the polymer network, significantly increasing the Young’s modulus and improving the structural integrity of the coating under physiological loads. Biologically, CLG provides essential RGD (Arg-Gly-Asp) tripeptide sequences that promote cell adhesion, migration, and proliferation, effectively transforming a synthetic surface into a “living” bio-interface [46,47,48].

Despite extensive research on hydrogels, the specific combination of a PVP/PEG matrix with a TAX-CD complex and CLG reinforcement for the modification of titanium surfaces remains unexplored. This study aims to fill this gap by providing a comprehensive analysis of the physicochemical behavior of these systems. We report on the structural morphology (scanning electron microscopy, SEM), chemical interactions (Fourier Transform Infrared Spectroscopy, FTIR), and mechanical properties (DMA). Furthermore, we evaluate the stability and degradation of these materials in complex simulated environments, including simulated body fluid (SBF), phosphate-buffered saline (PBS), and artificial saliva (AS), to validate their potential for real-world clinical applications in orthopedics and dental implantology. To provide a comprehensive understanding of the developed system, the study was structured into three main stages: (i) preparation and characterization of the ceramic phase (HAp-Mg) and composite substrates, (ii) synthesis and modification of PVP/PEG-based hydrogels, and (iii) evaluation of the resulting hydrogel coatings and their functional performance.

## 2. Results

### 2.1. FTIR Analysis of Hydroxyapatite Modified with Magnesium Ions

**Figure 1:**
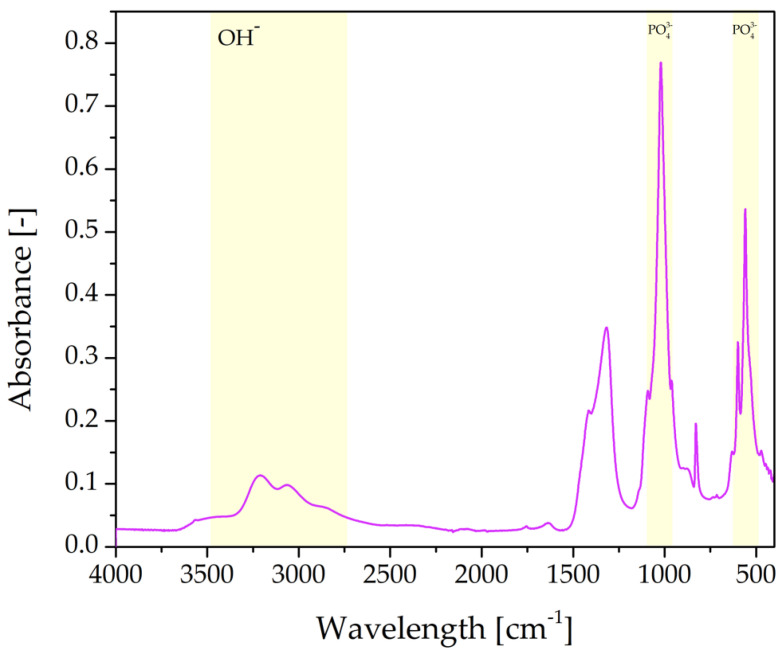
FTIR spectrum of magnesium-modified HAp. The highlighted regions indicate the characteristic absorption bands assigned to OH^−^ and PO_4_^3−^ groups

The FTIR spectrum of the obtained material (Figure 1) shows characteristic absorption bands corresponding to HAp. The presence of phosphate groups (PO_4_^3−^) is confirmed by bands in the range of approximately 560–605 cm^−1^ (bending vibrations ν_4_), around 960 cm^−1^ (symmetric stretching vibrations ν_1_), and in the range of 1000–1100 cm^−1^ (asymmetric stretching vibrations ν_3_). The observed bands are consistent with the literature data for HAp [49], confirming the formation of an apatite structure. Additionally, the presence of bands around 870 cm^−1^ and in the 1400–1500 cm^−1^ range is attributed to carbonate groups (CO_3_^2−^), indicating partial carbonate substitution in the apatite structure. This phenomenon is commonly observed in HAp obtained under atmospheric conditions and corresponds to the so-called type B substitution, in which CO_3_^2−^ ions replace phosphate groups [50]. A broad band in the 3000–3500 cm^−1^ range is associated with the presence of adsorbed water and hydroxyl groups. The presence of OH^-^ groups is characteristic of HAp. However, their identification (~3570 cm^−1^) may be hindered by band overlap and spectral noise, which is consistent with observations in nanocrystalline and modified materials [51]. The position and shape of the phosphate bands are consistent with the literature for HAp. However, their slight broadening and possible shifts may indicate reduced crystallinity or lattice deformations. Similar effects have been described for modified apatites, including those containing magnesium, but they may also result from the presence of carbonates and structural defects. Therefore, FTIR analysis does not allow for unequivocal confirmation of Mg^2+^ substitution. In summary, the FTIR spectrum confirms the preparation of HAp containing phosphate and carbonate groups, with properties similar to those of materials described in the literature. The observed broadening of phosphate-related bands and the presence of structural disorder are consistent with the XRD results, which also indicated reduced crystallinity and possible lattice distortions associated with magnesium modification.

### 2.2. X-Ray Diffraction of Magnesium-Modified Hydroxyapatite

X-ray diffraction (XRD) analysis confirmed that the obtained material had a structure characteristic of HAp. Phase identification was performed based on the ICDD reference card 01-080-7085 for HAp crystallizing in the hexagonal system (space group P63/m).

**Figure 2:**
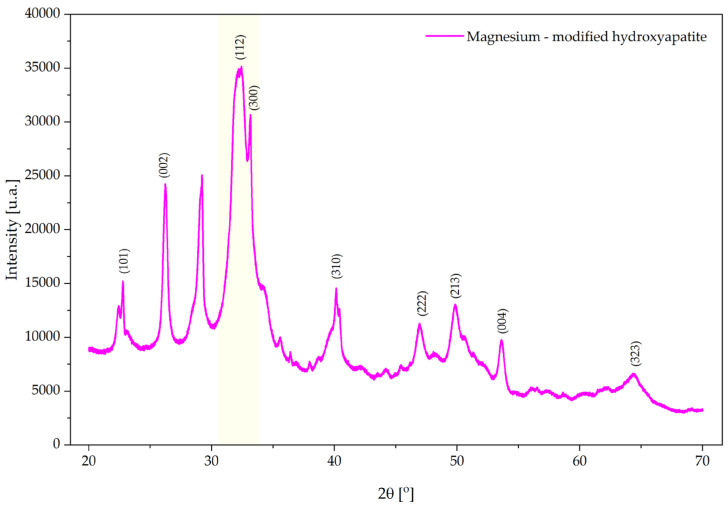
X-ray diffraction (XRD) pattern of the magnesium-modified HAp sample, along with a fit to the ICDD reference pattern. The visible agreement of the peaks confirms the presence of the apatite phase. The highlighted region indicates the characteristic reflections of HAp in the range of approximately 31–33° 2θ

Characteristic reflections attributed to HAp were observed in the diffractogram, corresponding to the crystallographic planes (101), (002), (112), (300), (310), (222), (213), (004), and (323), see Figure 2. Particularly characteristic of HAp is a set of reflections in the range of approximately 31–33° 2θ, corresponding to the (211)/(112)/(300) planes, considered the “fingerprint” of the HAp structure. The presence of these reflections confirms the preservation of the apatite structure after the magnesium modification process.

At the same time, a slight shift in some reflections toward larger 2θ values was observed relative to the positions reported for stoichiometric HAp in ICDD entry 01-080-7085. The most noticeable shifts concern the reflections associated with the (002), (310), (213), and (004) planes. These differences fall within the range of approximately 0.2–0.6° 2θ, which exceeds the typical instrumental error and may indicate local changes in the crystal lattice parameters.

This phenomenon may be associated with the partial substitution of Ca^2+^ ions by Mg^2+^ ions. Due to the smaller ionic radius of Mg^2+^ compared to Ca^2+^, the substitution of magnesium may lead to a local contraction of the crystal lattice, resulting in a decrease in interplanar distances and a shift in the reflections to larger diffraction angles in accordance with Bragg’s law. Similar effects, including shifts in diffraction peaks toward higher 2θ values, reductions in lattice parameters, and decreased crystallinity, have been reported previously for magnesium-substituted HAps and were attributed to the incorporation of Mg^2+^ ions into the apatite lattice [52].

Additionally, a partial broadening of the reflections is observed, particularly in the 31–33° 2θ range, which may indicate a decrease in crystallinity, a reduction in crystallite size, and the presence of lattice stresses induced by the presence of Mg^2+^ ions. This type of effect is frequently observed for magnesium-modified HAps described in the literature.

It should be emphasized, however, that XRD analysis alone does not allow for unequivocal confirmation of the complete and stoichiometric incorporation of Mg^2+^ ions into the HAp structure. Nevertheless, systematic shifts in the peaks, changes in peak shape, and peak broadening indicate a clear influence of magnesium modification on the crystalline structure of the obtained material. The results thus indicate that magnesium modification influenced the crystalline structure of HAp. The observed peak shifts and broadening are consistent with effects reported for magnesium-containing HAps, although they do not constitute direct proof of Ca^2+^/Mg^2+^ substitution within the apatite lattice [52,53].

In summary, XRD analysis confirms the predominance of the HAp phase with a hexagonal structure and the absence of detectable magnesium-containing crystalline secondary phases. The results may suggest the incorporation of magnesium into the material. However, unequivocal confirmation of Mg^2+^ substitution within the apatite lattice would require additional characterization techniques.

The obtained results indicate that the synthesized HAp is characterized by a nanocrystalline structure and a moderate degree of crystallinity. The average crystallite size determined using the Scherrer equation was approximately 9.80 nm, which confirms the formation of nanosized apatite crystallites. Such crystallite dimensions are characteristic of nano HAp materials and are often associated with an increased specific surface area and enhanced biological activity.

The calculated degree of crystallinity (Table 1) suggests a partially ordered apatite structure with reduced crystallinity compared to highly crystalline stoichiometric HAp. This result is consistent with the observed broadening of diffraction peaks in the XRD pattern and may be related to the presence of structural defects, carbonate substitution, or possible magnesium incorporation into the material. Reduced crystallinity is frequently reported for biologically inspired or ion-modified HAps and may positively influence their bioresorbability and biological performance.

### 2.3. SEM with Elemental Composition

#### 2.3.1. Hydroxyapatite

**Figure 3:**
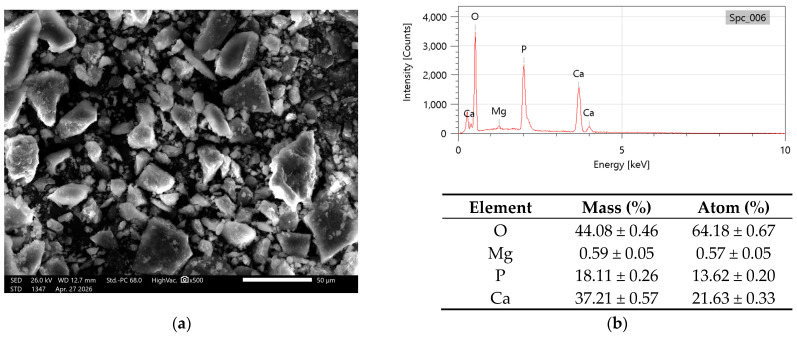
SEM–EDS analysis of magnesium-modified HAp: (**a**) SEM image showing irregular and agglomerated particles; (**b**) EDS spectrum confirming the presence of Ca, P, O, and Mg with elemental composition (wt% and at%).

Morphological analysis conducted using SEM revealed that the magnesium-modified HAp obtained exhibits an irregular particle structure and a marked tendency to form agglomerates. The SEM image (Figure 3) shows the presence of both larger, irregular fragments of varying shapes and finer grains distributed among them. This morphology indicates a non-uniform particle size distribution and a developed, rough surface of the material.

**Figure 4:**
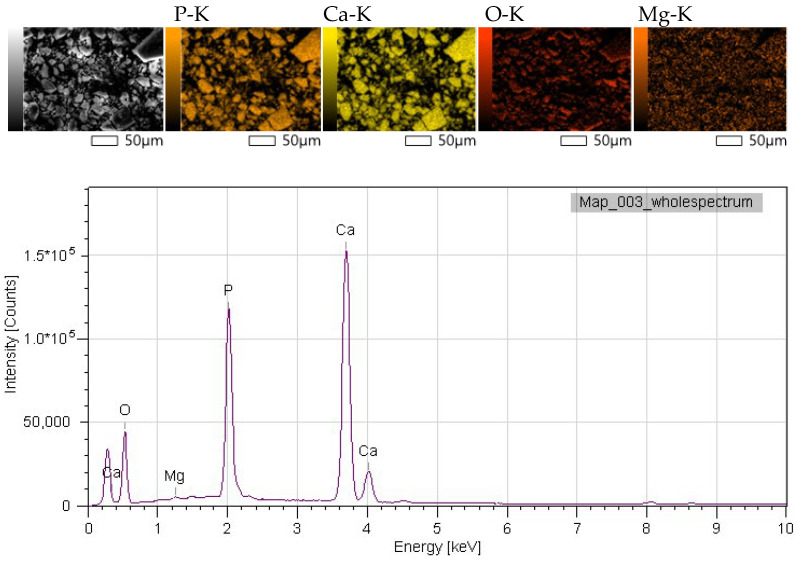
Elemental mapping images showing the distribution of P, Ca, O and Mg.

EDS analysis confirms the presence of elements characteristic of HAp, i.e., calcium (Ca) and phosphorus (P), and also reveals the presence of magnesium (Mg), indicating its inclusion in the material’s composition. The Ca/P atomic ratio calculated from EDS data was approximately 1.59, which is slightly lower than the stoichiometric value of 1.67 for HAp, suggesting possible calcium deficiency and/or partial substitution within the apatite structure. It should be noted that EDS provides only semi-quantitative results; therefore, the calculated ratio should be treated as an approximation. Elemental mapping (Figure 4) suggests a relatively uniform distribution of Mg in the examined area.

In combination with the results of the XRD analysis, which did not reveal the presence of distinct magnesium-containing phases, the obtained data suggest that magnesium does not occur as a separate crystalline phase but may be associated with the HAp material.

It should be noted, however, that EDS analysis is semi-quantitative and does not allow for an unambiguous determination of the degree of Mg^2+^ substitution in the HAp structure.

#### 2.3.2. Ti6Al4V/HAp-Mg Composite

An analysis of the microstructure of the Ti6Al4V/HAp composite performed using scanning electron microscopy (SEM) revealed the presence of two distinctly different phases corresponding to the material components. The tested material is a composite containing 5 wt% HAp and 5 wt% carboxymethylcellulose (CMC) in a Ti6Al4V alloy matrix.

**Figure 5:**
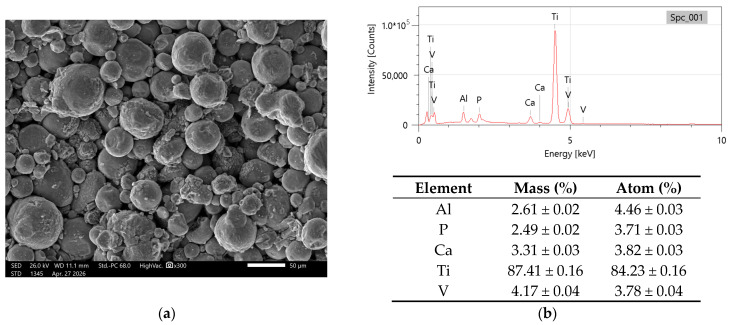
Microstructure and elemental analysis of Ti6Al4V/HAp-Mg composite: (**a**) SEM image showing spherical titanium alloy particles and finer irregular HAp particles; (**b**) EDS spectrum and quantitative composition confirming the presence of Ti, Al, V, Ca, and P.

The SEM image (Figure 5a) shows spherical Ti6Al4V alloy particles of varying sizes, characteristic of powders obtained by the atomization method. Among them, finer, irregular particles are observed, which can be attributed to the HAp phase. The microstructure indicates a non-uniform distribution of the composite components, with HAp particles dispersed in the spaces between titanium grains, while also exhibiting a tendency toward local agglomeration.

The presence of CMC, used as an auxiliary component in the forming process, may influence the distribution of particles and the degree of their aggregation. However, this is not directly observable in the SEM image after sintering.

EDS analysis (Figure 5b) confirmed the presence of elements characteristic of both phases of the composite. The spectrum is dominated by titanium (Ti) signals, which results from its volume predominance in the material, while the presence of aluminum (Al) and vanadium (V) confirms the use of the Ti6Al4V alloy. At the same time, the detection of calcium (Ca) and phosphorus (P) indicates the presence of a HAp phase in the composite structure. Magnesium was not detected in the EDS spectrum, which may be related to its low concentration resulting from the use of a small amount of Mg during HAp modification, as well as the detection limitations of the EDS technique for light elements present in minor amounts. Additionally, heterogeneous Mg distribution within the composite and possible redistribution during sintering cannot be excluded. Nevertheless, XRD analysis indicated structural changes consistent with the influence of magnesium modification on the HAp phase.

The results obtained confirm the presence of both main components of the composite and their coexistence within the material, which is consistent with the intended composition and the method of its production.

### 2.4. Surface Wettability

The surface properties of the Mg-modified Ti6Al4V/HAp composite were evaluated by measuring the contact angle using the hanging drop method. Water was used as the test liquid. The results are presented in Table 2.

Based on image analysis of the droplet, the contact angles on the left and right sides of the droplet were determined to be 52.19° and 56.11°, respectively. The average contact angle was 54.15°. The obtained contact angle value indicates the hydrophilic nature of the tested composite’s surface (θ < 90°). The presence of the HAp phase and its magnesium modification may enhance surface wettability, which is advantageous for potential biomedical applications, particularly for cell adhesion and integration with bone tissue.

### 2.5. Incubation Analysis in Simulated Fluids

The incubation analysis conducted in SBF, PBS, and AS demonstrated high chemical and structural stability of the investigated PVP, PEG, and MIX hydrogel systems over a 14-day period. In the SBF and PBS environments, pH values remained within the physiological range of 7.2–7.8. However, an initial decrease in pH in PEG-based systems suggests adaptation processes or the leaching of unreacted monomers, followed by subsequent stabilization of parameters. In AS, a lower initial pH (approx. 6.0) was recorded, which gradually increased to 6.6- a trend particularly evident in CLG-modified samples due to the presence of amino groups that stabilize the environment. Electrical conductivity measurements showed an increase during the first 4 days of incubation, indicating the diffusion of free ions and low-molecular-weight fractions into the medium, followed by the attainment of ionic equilibrium.

#### 2.5.1. pH Measurements

**Figure 6:**
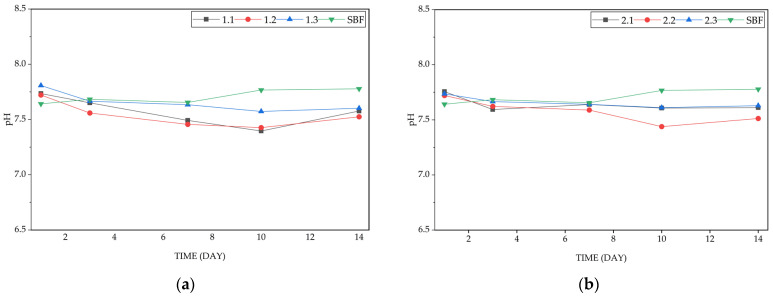
Results of incubation analysis using the following materials: (**a**) 1.1 PVP, 1.2 PEG, 1.3 MIX, (**b**) 2.1 PVP + CLG, 2.2 PEG + CLG, 2.3 MIX + CLG incubated in SBF.

**Figure 7:**
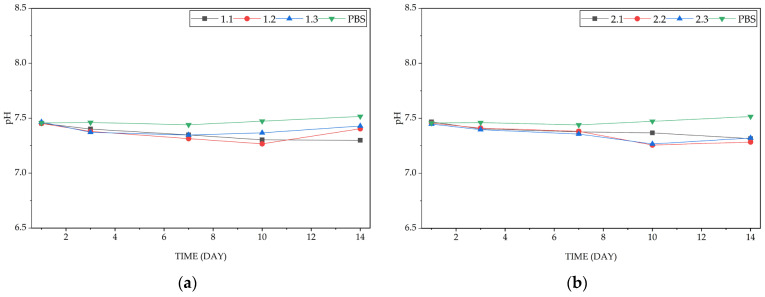
Results of incubation analysis using the following materials: (**a**) 1.1 PVP, 1.2 PEG, 1.3 MIX, (**b**) 2.1 PVP + CLG, 2.2 PEG + CLG, 2.3 MIX + CLG incubated in PBS.

**Figure 8:**
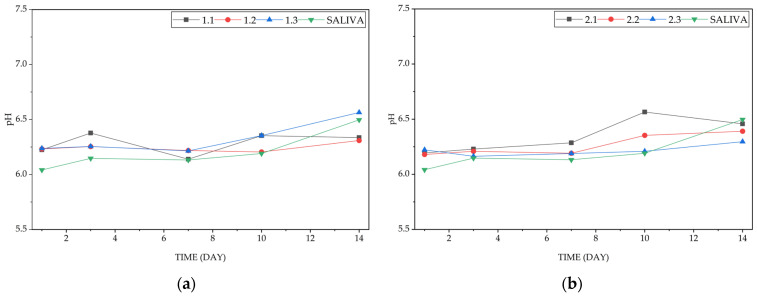
Results of incubation analysis using the following materials: (**a**) 1.1 PVP, 1.2 PEG, 1.3 MIX, (**b**) 2.1 PVP + CLG, 2.2 PEG + CLG, 2.3 MIX + CLG incubated in AS.

The pH variations for the PVP, PEG, and MIX-based materials, both with and without CLG modification, are presented in Figure 6, Figure 7 and Figure 8.

The pH values for all tested samples remained within the physiological range (approximately 7.4–7.8) throughout the incubation period. During the initial phase (days 1–7), a slight decrease in pH was observed for most materials, particularly for PEG-containing systems. This effect may be associated with the release of residual low-molecular-weight components, swelling-related processes, or partial hydrolytic changes occurring within the polymer network. In the later stages of incubation (days 11–14), the pH values tended to stabilize or showed a slight increase. CLG-containing materials exhibited similar trends with less pronounced fluctuations, which may suggest buffering interactions between CLG functional groups and the surrounding medium.

In PBS, the pH values remained within a relatively narrow range (7.2–7.5) for all investigated materials. Minor pH decreases observed during the first days of incubation for PEG and MIX samples were followed by stabilization during prolonged incubation. The addition of CLG did not significantly affect the overall pH profile of the tested systems.

In AS, the recorded pH values were lower (approximately 6.0–6.6), reflecting the acidic character of this incubation medium. A gradual increase in pH was observed over time, particularly after days 11 and 14. This effect was slightly more pronounced for CLG-containing samples and may be related to interactions between CLG amino groups and the incubation environment. Overall, the observed pH changes suggest relatively stable physicochemical behavior of the investigated hydrogels during incubation, while indicating ongoing interactions between the polymer network and the surrounding media.

#### 2.5.2. Electrical Conductivity Measurements

**Figure 9:**
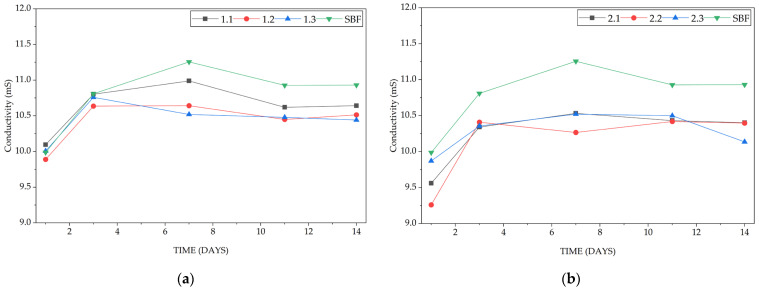
Results of incubation analysis using the following materials: (**a**) 1.1 PVP, 1.2 PEG, 1.3 MIX, (**b**) 2.1 PVP + CLG, 2.2 PEG + CLG, 2.3 MIX + CLG incubated in SBF.

**Figure 10:**
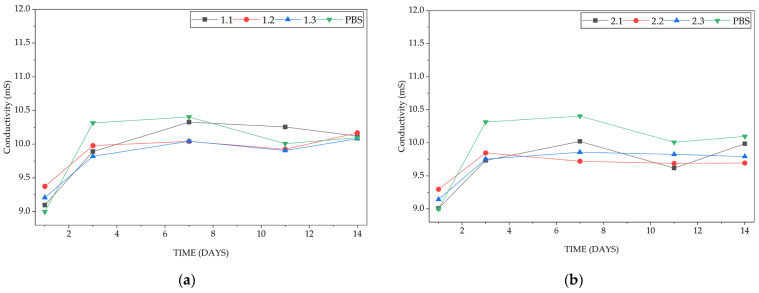
Results of incubation analysis using the following materials: (**a**) 1.1 PVP, 1.2 PEG, 1.3 MIX, (**b**) 2.1 PVP + CLG, 2.2 PEG + CLG, 2.3 MIX + CLG incubated in PBS.

**Figure 11:**
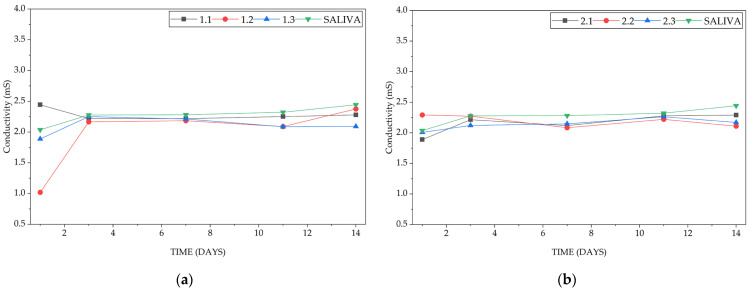
Results of incubation analysis using the following materials: (**a**) 1.1 PVP, 1.2 PEG, 1.3 MIX, (**b**) 2.1 PVP + CLG, 2.2 PEG + CLG, 2.3 MIX + CLG incubated in AS.

The monitoring of electrical conductivity (Figure 9, Figure 10 and Figure 11) served as an indicator of ion release and structural integrity of the hydrogels.

In the SBF and PBS environments, a noticeable increase in electrical conductivity was observed during the initial incubation period (days 1–4), which may be associated with the diffusion of mobile ions and low-molecular-weight components from the hydrogel network into the surrounding medium. In the subsequent stages of incubation, the conductivity values tended to stabilize or showed only minor fluctuations, which may indicate a reduction in the intensity of ion-exchange processes over time.

In contrast, the AS environment exhibited the lowest conductivity values throughout the study. A gradual increase in conductivity was observed after the seventh day of incubation with no abrupt changes, suggesting relatively limited release of ionic species into the medium.

CLG-containing materials exhibited conductivity profiles generally comparable to those of the corresponding base polymer systems, though fluctuations were slightly smaller in some cases. This behavior may suggest that CLG influences interactions occurring between the hydrogel matrix and the incubation medium.

Overall, the obtained conductivity results suggest gradual ion exchange processes and interactions between the hydrogel matrices and the surrounding media during incubation.

### 2.6. Mass Changes in Hydrogels During Incubation

**Figure 12:**
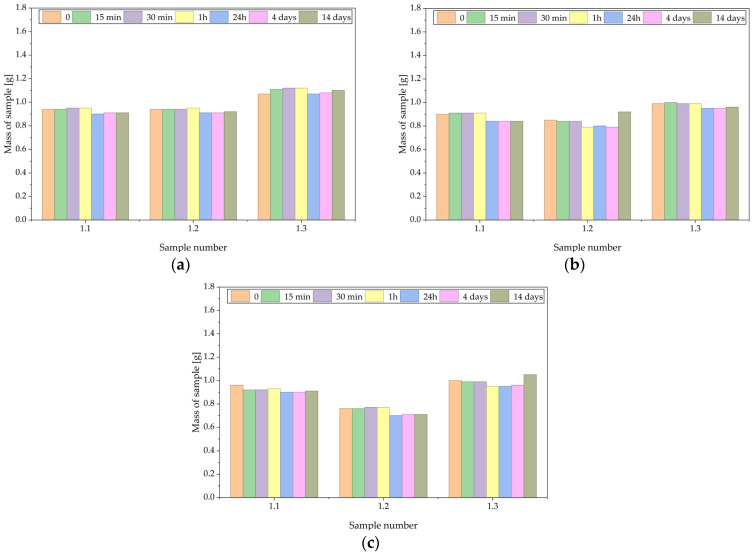
Degradation behavior of hydrogel materials: 1.1 (PVP), 1.2 (PEG), and 1.3 (MIX) in different incubation media: (**a**) SBF, (**b**) phosphate-buffered saline (PBS), and (**c**) AS.

**Figure 13:**
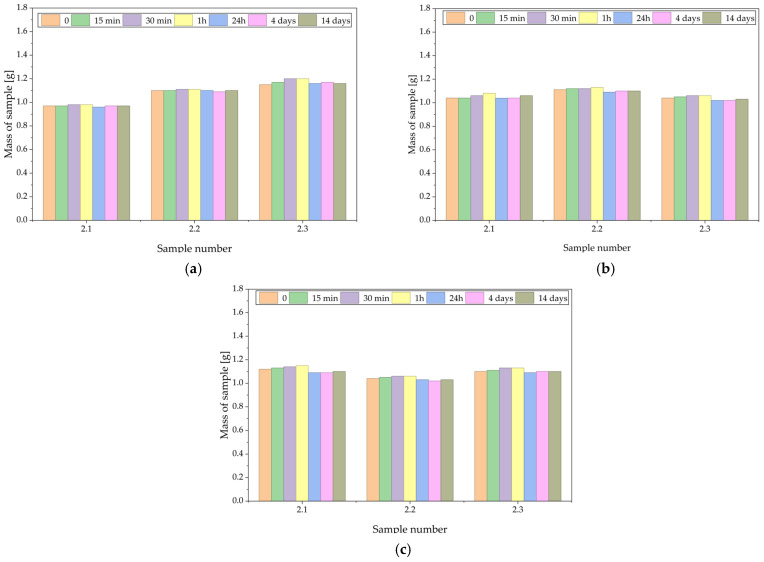
Degradation behavior of CLG-containing hydrogel materials: 1 (PVP + CLG), 2 (PEG + CLG), and 3 (MIX + CLG) in different incubation media: (**a**) SBF, (**b**) PBS, and (**c**) AS.

Figure 12 and Figure 13 present the mass changes in hydrogel materials based on PVP, PEG, and their mixture (MIX), as well as the corresponding CLG-containing systems (2.1–2.3), during incubation in SBF, phosphate-buffered saline (PBS), and AS. The measurements were performed before incubation and after 15 min, 30 min, 1 h, 24 h, 4 days, and 14 days of incubation. For the CLG-free hydrogels (Figure 12), only relatively small mass variations were observed during the incubation period. Depending on the incubation medium and hydrogel composition, both slight increases and decreases in sample mass were recorded. These effects may be associated with simultaneous liquid uptake, swelling-related processes, and partial release of low-molecular-weight components from the hydrogel matrix. With prolonged incubation, moderate decreases in mass were observed in selected samples, particularly in PBS and AS, indicating gradual physicochemical changes occurring within the polymer structure during incubation. Among the investigated systems, PEG- and MgI-based materials generally exhibited slightly greater mass changes than PVP hydrogels, whereas samples incubated in SBF showed the smallest changes over time. This may suggest relatively weaker interactions between the hydrogel matrices and the SBF environment compared with PBS and AS. A similar tendency was observed for CLG-containing materials (Figure 13, samples 2.1–2.3). However, in several cases the magnitude of mass changes was slightly lower than in the corresponding CLG-free systems, which may indicate that the presence of CLG influenced the incubation behavior of the hydrogels. The most noticeable mass variations were again observed in PBS and AS, while samples incubated in SBF exhibited relatively minor changes throughout the experiment.

Overall, the obtained results indicate relatively small mass changes during incubation, suggesting limited physicochemical changes within the investigated hydrogel systems under the applied experimental conditions. The observed variations most likely reflect overlapping processes occurring during incubation, including swelling, diffusion of soluble components, and gradual interactions between the hydrogel matrices and the surrounding media.

### 2.7. SEM Analysis

SEM was employed to evaluate the surface morphology of the synthesized hydrogels based on PVP, PEG, and their blends (MIX). The analysis also focused on the structural impact of CLG incorporation, incubation in SBF, and modification with TAX.

#### 2.7.1. Morphology of Base Polymer Matrices

**Figure 14:**
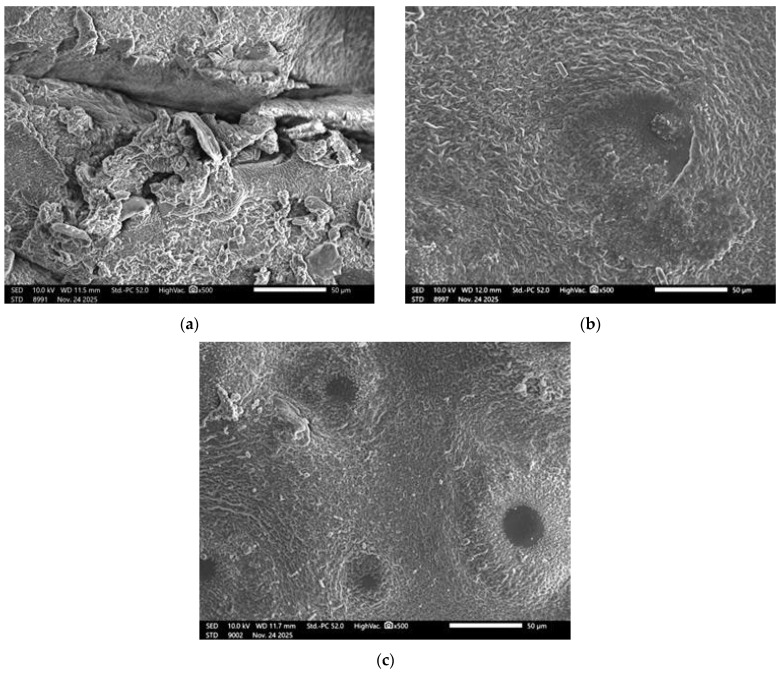
Surface morphology of the materials: (**a**) PVP, (**b**) PEG, (**c**) PVP + PEG at 500× magnification.

The surface of the pure PVP hydrogel (Figure 14a) observed at 500× magnification is characterized by a relatively homogeneous and dense morphology with a distinctly folded topography. The irregular surface folds are typical of amorphous polymeric materials and may be associated with shrinkage occurring during sample drying. No large pores or major surface defects were observed within the analyzed area. In contrast, the PEG hydrogel (Figure 14b) exhibited a more heterogeneous morphology with local irregularities, depressions, and regions of varying texture. Compared with the PVP system, the PEG matrix appeared less uniform, with visible variations in surface morphology. The blended PVP + PEG hydrogel (Figure 14c) showed an intermediate morphology between the single-component systems. The surface remained relatively uniform but contained fine pits and localized irregularities, which may indicate relatively good dispersion of both polymer components within the hydrogel matrix.

A comparative analysis of the base polymer matrices indicates that the PVP hydrogel exhibited the most homogeneous surface morphology, whereas the PEG-based material showed greater surface heterogeneity. The PVP/PEG blend demonstrated intermediate characteristics with improved uniformity compared to the PEG system. The increased surface heterogeneity observed after incubation may also indicate ongoing interactions between the hydrogel matrix and ions present in SBF. However, direct confirmation of apatite precipitation or bioactive mineralization would require complementary compositional analyses.

#### 2.7.2. Impact of Collagen Incorporation

**Figure 15:**
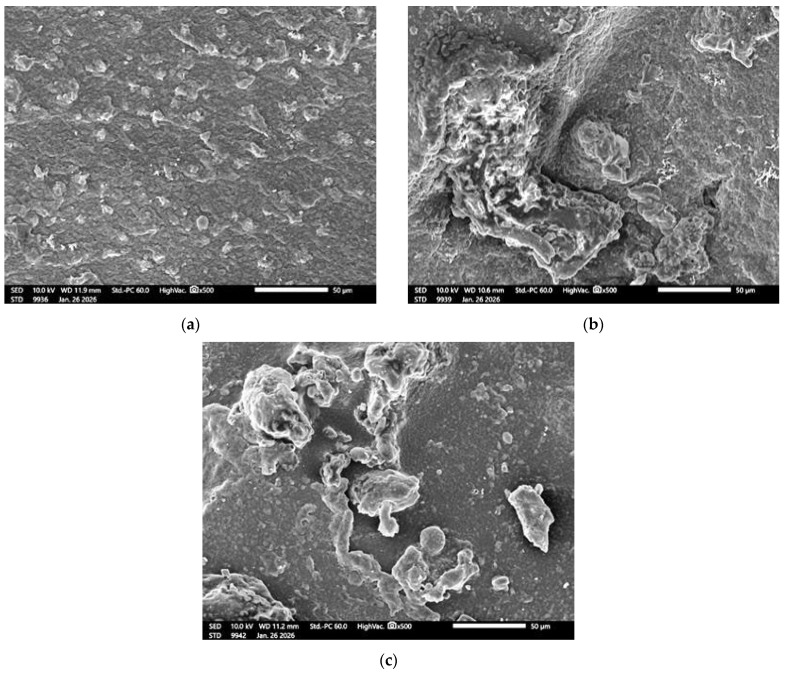
Surface morphology of the materials: (**a**) PVP + CLG, (**b**) PEG + CLG, (**c**) PVP + PEG + CLG at 500× magnification.

The incorporation of CLG visibly influenced the morphology of the investigated materials (Figure 15). In the PVP + CLG system (Figure 15a), the surface became rougher, with irregular agglomerate-like structures appearing on the polymer background. The PEG + CLG material (Figure 15b) exhibited a more complex and irregular morphology, including fibrillar-like structures that may be associated with CLG-rich regions. The PVP + PEG + CLG blend (Figure 15c) showed the most developed surface morphology, characterized by numerous irregular agglomerates and surface precipitate-like features. Compared with the unmodified matrices, CLG-containing systems generally exhibited increased roughness and more heterogeneous surface organization. However, the SEM observations should be interpreted primarily as qualitative morphological characterization of dried hydrogel materials.

#### 2.7.3. Surface Evolution Post-SBF Incubation

**Figure 16:**
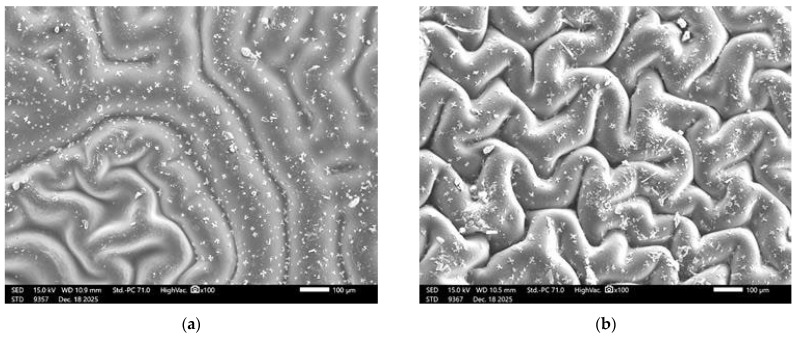
Surface morphology of the materials after incubation in SBF: (**a**) PVP + PEG, (**b**) PVP + PEG + CLG at 100× magnification.

The morphology of the investigated materials was additionally affected by incubation in SBF (Figure 16). After incubation, the PVP + PEG system exhibited folded surface structures, with fine particles distributed across the matrix surface. Similar features were observed for the PVP + PEG + CLG material, although the particulate structures appeared more pronounced. The bright crystalline-like deposits visible on the surfaces may originate from inorganic salts present in the SBF medium that crystallized during sample drying and preparation. Compared with the non-incubated samples, the incubated materials exhibited increased surface heterogeneity and additional particulate features. However, based solely on SEM observations, unequivocal confirmation of mineral phase formation or surface mineralization is not possible and would require complementary analyses such as EDS, FTIR, or XRD performed after incubation.

#### 2.7.4. Taxifolin Modification

**Figure 17:**
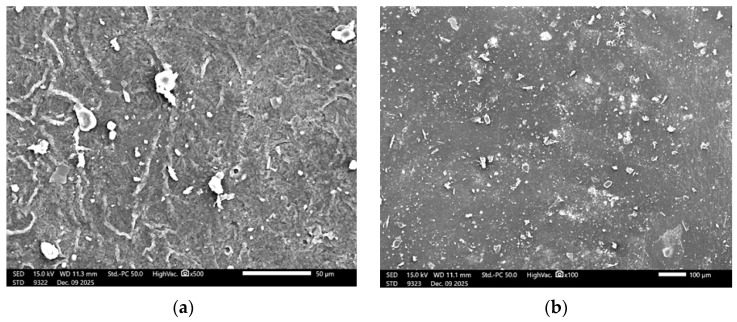
Microstructure of the (**a**) PVP + PEG + βCD-TAX material and (**b**) the PVP + PEG material modified with TAX through incubation in 1 mL of TAX solution.

Regarding bioactive modifications, the PVP + PEG system modified with the βCD-TAX complex (Figure 17a) exhibited a relatively homogeneous surface morphology, although fine particles, potentially associated with residual PBS salts or dried surface deposits, remained visible. Similarly, the post-loading method involving 1 mL of TAX (Figure 17b) resulted in the appearance of fine surface structures without visible macroscopic changes in the overall morphology of the hydrogel matrix. The obtained SEM observations suggest that TAX incorporation, both through βCD complexation and the post-loading approach, did not markedly alter the general surface morphology of the investigated hydrogel systems. Nevertheless, the presented observations should be interpreted as qualitative morphological characterization of the modified hydrogel surfaces.

### 2.8. FTIR Analysis

**Figure 18:**
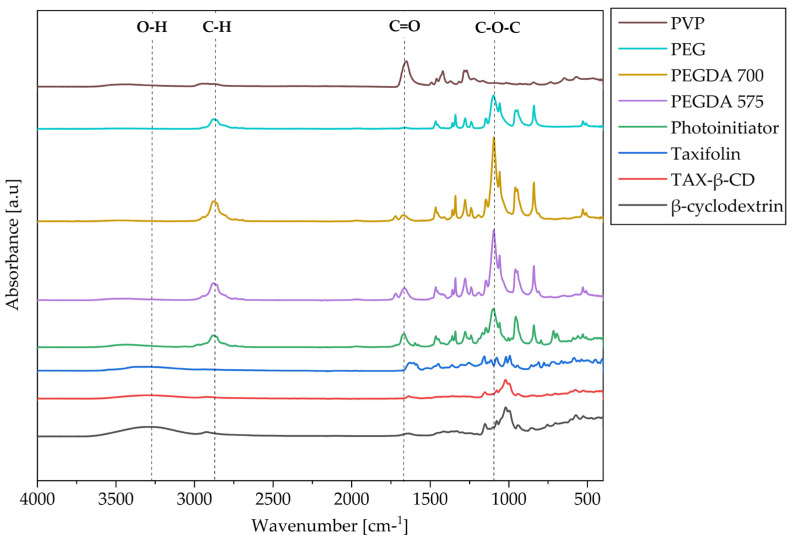
FTIR spectra of the components used during the synthesis of biomaterials.

**Figure 19:**
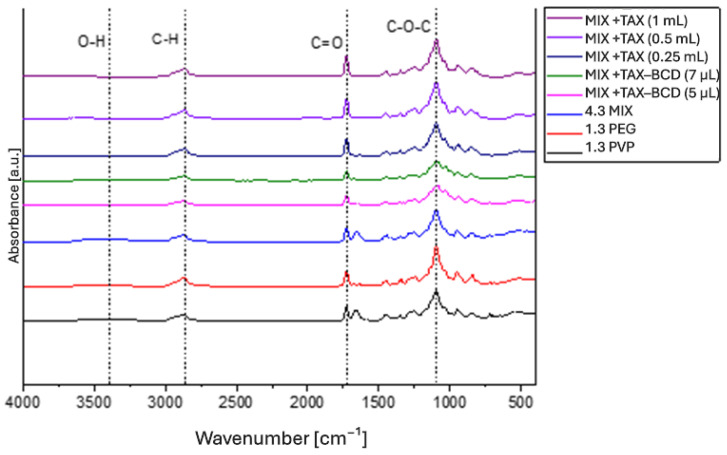
FTIR spectra of the obtained hydrogel materials.

The chemical structure of the raw components and the resulting hydrogel materials was characterized using FTIR spectroscopy. The FTIR spectra of the raw components used for hydrogel synthesis are presented in Figure 18, while the spectra of the obtained hydrogel materials are shown in Figure 19. The spectra of the cross-linked hydrogels with varying chemical compositions are shown in Figure 19. A comparative analysis of both sets of spectra allows for the identification of characteristic functional groups and an assessment of the cross-linking process’s impact on the materials’ final chemical structure.

In the wavenumber range of 3200–3600 cm^−1^, both the raw components and the resulting hydrogels exhibit broad absorption bands corresponding to the stretching vibrations of hydroxyl groups (-OH). These bands are particularly intense for βCD and TAX, reflecting numerous hydrogen bonds, typical of polysaccharides and phenolic compounds. In the 2850–3000 cm^−1^ region, absorption bands corresponding to the CH stretching vibrations of aliphatic groups are observed. These are characteristic of the polymer chains in PEGDA and PVP and are present in both the precursors and the final hydrogels, confirming that the aliphatic structure of the polymer segments remains intact after cross-linking. Distinct bands in the range of 1700–1750 cm^−1^ are attributed to the stretching vibrations of carbonyl groups (C=O). These signals are characteristic of the ester groups in PEGDA and the amide groups in PVP. Furthermore, the interval between 1000 and 1300 cm^−1^ contains intense bands corresponding to C-O-C stretching vibrations. These are typical of the ether structures in PEG and the glucopyranose rings of the CD molecule. A comparison between the FTIR spectra of the starting components and the synthesized hydrogel materials reveals no new absorption bands. This suggests that the incorporation of additives, such as TAX and βCD, occurs primarily through physical interactions rather than the formation of new covalent bonds with the polymer matrix.

In addition, PEGDA 575/700 served as the photo-curable component of the hydrogel due to the presence of terminal acrylate groups bearing reactive C=C double bonds. During UV irradiation, the photoinitiator generated free radicals that initiated photopolymerization and crosslinking of PEGDA, leading to the formation of a three-dimensional hydrogel network. Although no new, unambiguous absorption bands appeared after UV curing, the observed slight changes in intensity and broadening of the bands associated with carbonyl and ether groups may indirectly indicate the formation of a cross-linked polymer structure. The FTIR results therefore support the occurrence of UV-induced PEGDA crosslinking and suggest that TAX and βCD were incorporated into the system primarily via physical interactions and hydrogen bonding rather than covalent bonding with the polymer matrix.

### 2.9. Taxifolin Release—Via UV–Vis Spectroscopy

**Figure 20:**
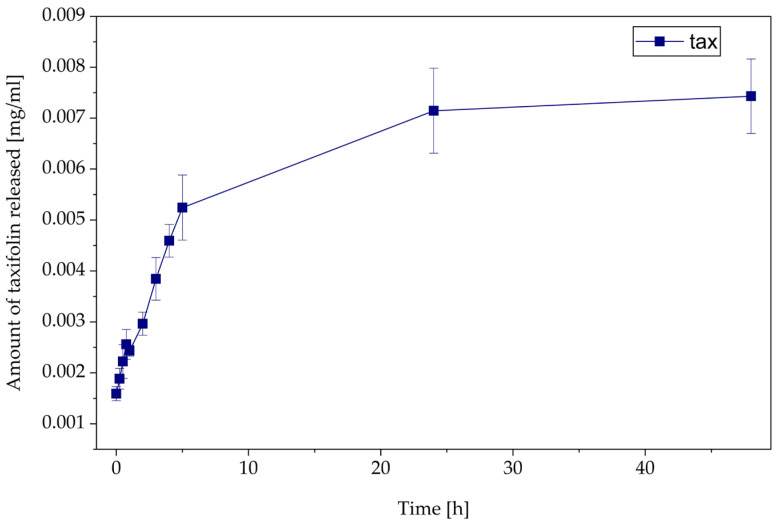
Time-dependent release profiles of TAX from TAX-β hydrogel materials determined by UV–Vis spectroscopy.

Figure 20 presents the release profile of TAX from the investigated hydrogel system during 48 h of incubation in PBS at 37 °C. A gradual increase in the amount of released TAX was observed over time, indicating sustained diffusion of the flavonoid from the polymer matrix. The most dynamic release occurred during the initial incubation period (0–6 h), suggesting the diffusion of TAX molecules located near or at the hydrogel surface. After 24 h, the release rate became less pronounced and approached a plateau, which may indicate progressive depletion of readily diffusible TAX fractions and increasing diffusion limitations within the hydrogel network. The obtained release profile suggests that TAX release was governed primarily by diffusion-related processes occurring within the crosslinked polymer matrix. The relatively gradual increase in the concentration of the released compound also indicates that the hydrogel system maintained structural integrity during incubation without rapid matrix disintegration. Error bars representing standard deviation demonstrate acceptable repeatability of the measurements performed in triplicate (n = 3).

### 2.10. Dynamic Mechanical Analysis

Dynamic mechanical analysis (DMA) was performed on PEG, PVP, PVP + PEG (MIX), and PVP + PEG + CLG samples to evaluate their mechanical properties and determine the influence of material composition on the stiffness of the studied systems. The DMA method is widely used in biomaterial characterization because it enables precise analysis of a material’s response to mechanical loading within the elastic range. Based on the experimental data, stress–strain curves were plotted. All analyzed samples exhibited an initial linear region characteristic of an elastic response, allowing determination of Young’s modulus via linear approximation. The calculated Young’s modulus values are summarized in Table 3.

The PEG sample exhibited the lowest Young’s modulus, indicating high elasticity and low material stiffness. This finding is consistent with the literature data describing PEG as a soft material capable of providing a good mechanical match to soft tissues. Both the PVP and PVP + PEG samples showed higher Young’s modulus values, pointing to increased stiffness resulting from stronger interchain interactions within the PVP network. Notably, the addition of PEG to the PVP matrix did not significantly soften the material. The highest Young’s modulus was recorded for the PVP + PEG + CLG sample, clearly demonstrating the reinforcing effect of CLG, which acts as a natural structural strengthening agent within the polymer matrix. These results confirm that the mechanical properties of the hydrogels, specifically stiffness and elasticity, can be easily tuned by adjusting the material composition. This tunability is crucial for biomedical applications, as it enables alignment of mechanical properties with specific target tissues, thereby minimizing the risk of mechanical mismatch between the implant and the surrounding biological environment.

**Figure 21:**
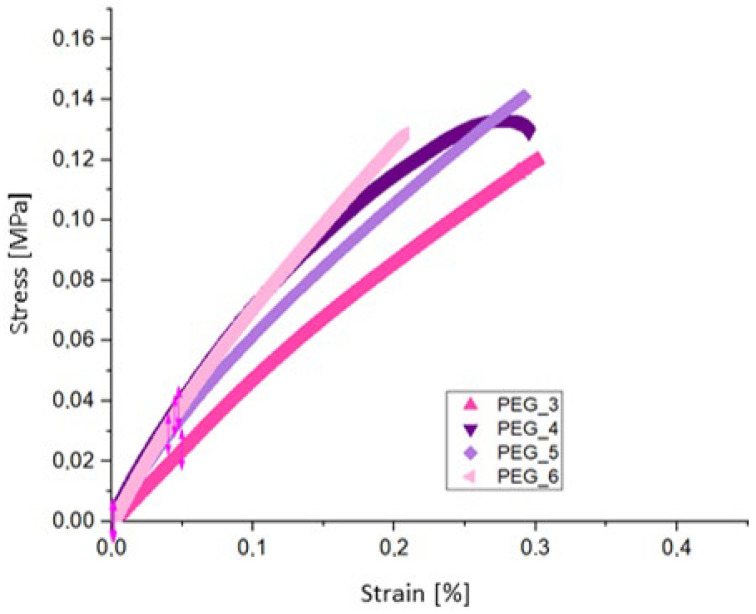
Analysis of the stress–strain relationship for a PEG specimen in four replicates.

**Figure 22:**
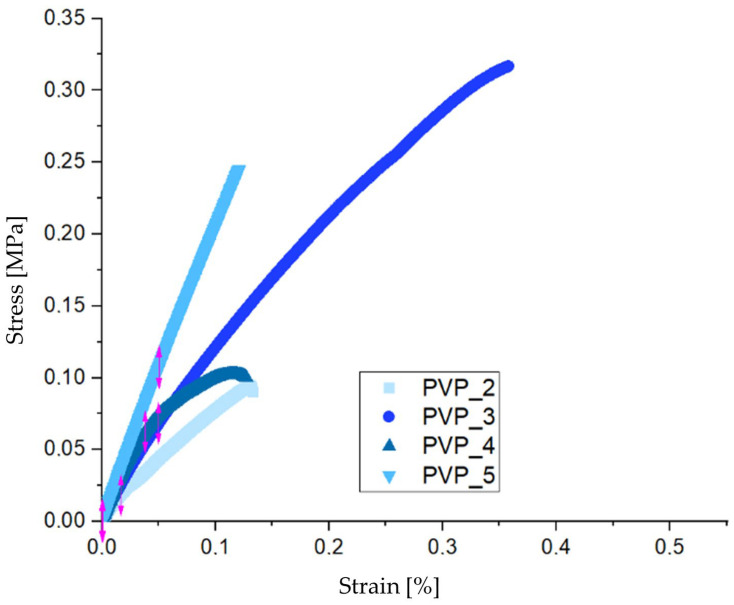
Analysis of the stress–strain relationship for a PVP specimen in four replicates.

**Figure 23:**
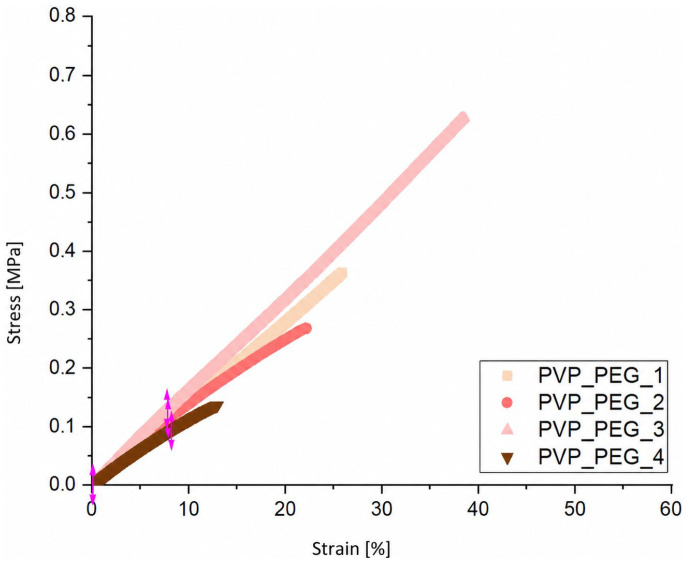
Analysis of the stress–strain relationship for a PVP + PEG specimen in four replicates.

**Figure 24:**
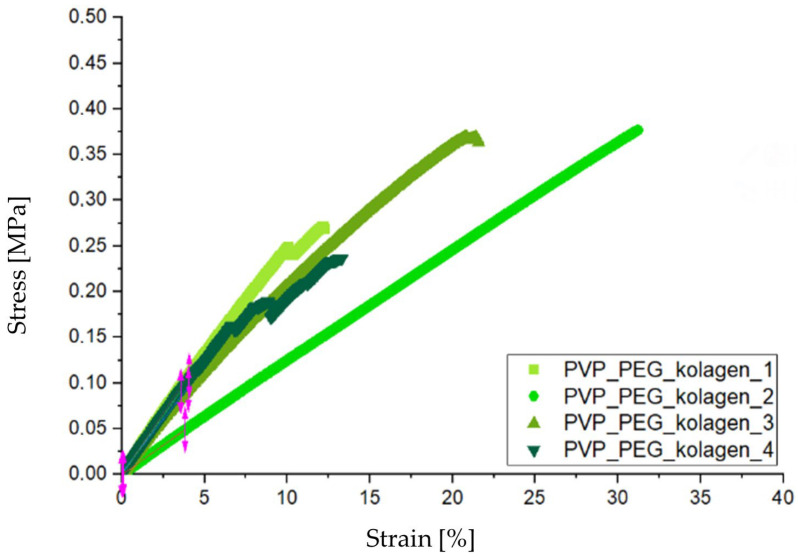
Analysis of the stress–strain relationship for a PVP + PEG + CLG specimen in four replicates.

Representative stress–strain curves for all analyzed hydrogel systems are presented in Figure 21, Figure 22, Figure 23 and Figure 24. The pink arrows indicate the initial linear region of the stress–strain curves, which was used to determine the Young’s modulus of the investigated materials. In all cases, an initial linear-deformation region was observed and used to determine the Young’s modulus. PEG-based materials exhibited the lowest stress values, whereas CLG-containing systems showed the highest stiffness, confirming the reinforcing effect of CLG within the hydrogel matrix.

### 2.11. Indirect Cytotoxicity Test

The evaluation of cytotoxic properties in biomaterials designed for bone regeneration is an essential step in determining their biological safety and suitability for implantation. Performing extract-based analysis enables comprehensive assessment of cellular responses by distinguishing the influence of substances released from the material. This plays a crucial role in regulating cell survival, proliferation, and functionality within the bone defect environment [54].

To evaluate the biological safety of the developed materials, indirect cytotoxicity studies were conducted in accordance with ISO 10993-5 [55] recommendations. Material extracts were obtained by incubating the samples in α-MEM culture medium for 24 h [56]. Subsequently, MC3T3-E1 preosteoblasts, which play an important role in bone formation and osteogenic differentiation, were exposed to the prepared extracts for an additional 24 h.

**Figure 25:**
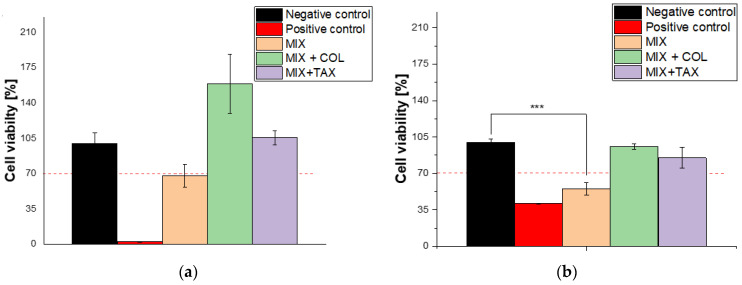
Results of indirect cytotoxicity test based on (**a**) XTT reduction; (**b**) resazurin assay. The red dashed line indicates the 70% cell viability threshold, below which the material is considered cytotoxic according to ISO 10993-5.The results are statistically significant when *** *p* < 0.001.

Cellular responses were analyzed using two complementary viability assays. The XTT assay (XTT tetrazolium salt assay) (Figure 25a) was used to assess mitochondrial metabolic activity, whereas the resazurin assay (Figure 25b), which measures the conversion of resazurin into resorufin by metabolically active cells, was used to evaluate overall cell viability and metabolic function. According to ISO 10993-5 criteria, materials that decrease cell viability by 30% or more compared with the control group are considered cytotoxic and therefore unsuitable for biomedical applications involving direct contact with tissues and physiological fluids. The results from both assay types are consistent. The MIX material, based on PVP and PEG and crosslinked using PEGDA 575 and a photoinitiator, exhibited cytotoxic effects in both tests. Despite the material preparation procedure, which included rinsing the samples in PBS, it was not possible to completely remove residual toxic reagents used during fabrication. A similar relationship was reported by Liu et al., who demonstrated that the cytotoxicity of materials may depend strongly on the degree of purification and on the presence of low-molecular-weight compounds released during incubation. The authors emphasized that a sufficiently long washing or conditioning process is crucial for preparing materials for biological studies, as it removes potentially toxic residues [57]. Interestingly, this unfavorable effect was eliminated by modifying the composition of the prepared materials. The incorporation of CLG significantly improved the cytocompatibility of the materials. In both the XTT and Resazurin assays, cytotoxicity levels of 158.99 ± 29.06% and 95.93 ± 2.62% were observed, respectively. This effect is associated with the properties of CLG, which is a major component of the bone extracellular matrix, a highly organized structure composed not only of CLG proteins, but also of non-CLGous proteins and small proteoglycans, additionally reinforced by HAp nanocrystals [58]. Potahhara et al. demonstrated that materials composed of CLG/gelatin and HAp did not exhibit cytotoxic effects [59]. Similar findings were reported by Li et al. for materials based on human-like CLG and HAp. The results obtained are consistent with reports in the literature indicating that CLG-HAp scaffolds exhibit low cytotoxicity and high cytocompatibility, thereby promoting the adhesion, survival, and proliferation of bone cells [60]. Modification of the materials by incorporating the bioactive TAX-βCD complex also enabled the development of materials that, according to ISO 10993-5, did not exhibit cytotoxic effects. Cell viability relative to the negative control, determined using the XTT and Resazurin reduction assays, reached 105.52 ± 6.89% and 85.05 ± 10.21%, respectively. Viability values above 100% should also be interpreted with caution, especially for TAX-containing samples. Since TAX is an antioxidant flavonoid with redox activity, it may potentially influence the response of XTT/resazurin assays after being released from the βCD complex. For this reason, these values were considered primarily as an indication that the tested systems were not cytotoxic and showed good cytocompatibility, rather than as clear evidence of increased cell proliferation or metabolic stimulation. Nevertheless, the favorable cellular response observed for TAX-containing systems may be related to the known biological properties of TAX, including its anti-inflammatory and antioxidant activity, as well as its reported ability to inhibit osteoclastogenesis. Previous studies have shown that this compound reduces bone loss by suppressing osteoclast activity and modulating RANKL-dependent signaling pathways, indicating its considerable therapeutic potential in osteoporosis treatment [61]. Interesting studies confirming the significant potential of TAX were also presented by Jiang et al., who developed a drug delivery system based on hollow mesoporous whitlockite nanoparticles (MWHNPs) loaded with TAX and encapsulated within a GelMA hydrogel. The incorporation of TAX into the biomaterial simultaneously inhibited osteoclastogenesis, enhanced osteogenesis, and stimulated angiogenesis, ultimately improving the regeneration of bone defects under osteoporotic conditions [62].

## 3. Discussion

The results obtained in this study demonstrate that the developed PVP/PEG-based hydrogel systems can be successfully combined with titanium-based substrates to form multifunctional coatings with tunable physicochemical and mechanical properties. The applied approach integrates ceramic, metallic, and polymeric components into a single platform, enabling simultaneous modification of surface characteristics and the incorporation of bioactive functionality. Therefore, the interpretation of the obtained results should be considered as a sequence of mutually related effects, in which the substrate composition and surface morphology determine hydrogel coating formation, while polymer composition, CLG reinforcement, and TAX-βCD incorporation jointly influence stability, release behavior, and biological response.

The structural analyses confirmed the successful synthesis of HAp with features characteristic of apatite materials described in the literature. FTIR and XRD results revealed the presence of phosphate groups and reflections corresponding to the hexagonal HAp structure. At the same time, carbonate-related bands detected in the FTIR spectra indicate partial carbonate substitution, which is commonly observed in apatites synthesized under atmospheric conditions. Although the applied techniques do not allow for unequivocal confirmation of complete Mg substitution within the apatite lattice, the combined FTIR, XRD, and EDS results indicate structural modification of the apatite phase, reflected by carbonate-related bands, peak broadening, reduced Ca/P ratio, and the absence of detectable Mg-rich secondary crystalline phases. Nevertheless, the absence of secondary magnesium-containing phases suggests that magnesium may be associated with the apatite structure rather than occurring as a separate crystalline compound. The reduced Ca/P ratio additionally indicates the formation of calcium-deficient apatite, which is frequently associated with increased surface activity and higher reactivity in physiological environments.

SEM observations of the Ti6Al4V/HAp composite revealed a heterogeneous microstructure consisting of spherical metallic particles and irregular HAp agglomerates. Such morphology is characteristic of powder-based processing methods and confirms the coexistence of both phases within the composite structure. The presence of the ceramic phase may increase surface reactivity, while the metallic matrix ensures the mechanical stability required for potential implant-related applications. Importantly, this heterogeneous, ceramic-containing surface also provides favorable anchoring sites for the hydrogel layer, thereby linking the substrate architecture directly to the effectiveness of subsequent coating formation.

The morphology of the hydrogel systems was strongly dependent on polymer composition. PVP-based materials exhibited the most homogeneous surface structure, which may be related to the PVP’s favorable film-forming ability and stronger intermolecular interactions within the polymer network. In contrast, PEG-containing systems exhibited greater heterogeneity and local irregularities, likely due to higher chain mobility and lower structural cohesion. The mixed PVP/PEG systems demonstrated intermediate characteristics, indicating relatively good compatibility between the two polymers and the formation of a uniform hydrogel network without evident macroscopic phase separation. These morphological differences explain the later incubation and release behavior: more cohesive PVP-rich networks may restrict diffusion and mass loss, whereas PEG-containing systems, owing to their higher hydrophilicity and chain mobility, are more susceptible to water penetration, swelling, and gradual release of incorporated compounds.

The incorporation of CLG noticeably affected both the morphology and mechanical behavior of the hydrogels. SEM analysis revealed increased surface roughness and the appearance of fibrillar-like structures, which may result from local CLG aggregation within the synthetic matrix. Simultaneously, DMA demonstrated a significant increase in Young’s modulus for CLG-containing systems, indicating that CLG may act as a reinforcing component within the hydrogel structure. The observed increase in stiffness suggests restricted polymer chain mobility and additional intermolecular interactions introduced by the biopolymer. These findings confirm that the mechanical properties of the developed coatings can be effectively tailored through compositional modification.

The incubation studies performed in SBF, PBS, and AS demonstrated relatively stable physicochemical behavior of the investigated materials. The observed pH fluctuations remained within ranges considered acceptable for physiological conditions, while only moderate changes in conductivity were detected during incubation. PEG-rich systems exhibited slightly higher susceptibility to degradation and mass loss, which may be associated with their lower structural compactness and greater affinity for water uptake. This behavior correlates with the SEM observations, in which PEG-containing systems demonstrated greater structural heterogeneity, suggesting that less compact network organization facilitated fluid penetration and diffusion of low-molecular-weight components. In contrast, PVP-containing hydrogels showed improved structural stability during long-term incubation. The addition of CLG appeared to further stabilize the hydrogel matrices, as reflected by reduced degradation in the CLG-modified samples.

Surface observations following incubation revealed particulate deposits, particularly in CLG-containing systems exposed to SBF. These structures may be associated with the precipitation of inorganic species originating from the incubation medium, suggesting active interaction between the material surface and ions present in the surrounding environment. However, the contribution of residual salts from sample preparation cannot be completely excluded and should therefore be considered when interpreting the results.

An important aspect of the developed systems was the incorporation of TAX as a bioactive compound. Both the βCD-assisted incorporation method and the post-loading approach allowed for successful functionalization of the hydrogel matrices without disrupting their structural integrity. The gradual release profile of TAX observed in the present study may be directly associated with the internal organization of the hydrogel network and the physicochemical properties of the polymer matrix. The SEM analysis demonstrated that the PVP/PEG-based systems exhibited relatively compact yet heterogeneous surface morphologies, while FTIR analysis indicated numerous hydrogen-bonding interactions among the polymer components, βCD, and TAX. Such interactions may contribute to temporary retention of the flavonoid within the hydrogel structure thereby slowing down its diffusion into the surrounding medium. Additionally, the relatively limited total amount of released TAX and the absence of an abrupt burst-release effect suggest that the crosslinked polymer matrix maintained structural stability during incubation. This interpretation remains consistent with the degradation and incubation studies, where only minor mass changes and relatively stable conductivity and pH values were observed throughout the experiment. Together, these findings indicate that the investigated hydrogels undergo gradual swelling and controlled diffusion rather than rapid matrix degradation. The release behavior may also be influenced by the presence of PEG within the polymer system. Due to its hydrophilic character and high chain mobility, PEG likely facilitates water penetration into the hydrogel network, thereby promoting diffusion of TAX molecules. Simultaneously, the presence of PVP may contribute to the formation of denser polymer domains through intermolecular interactions, limiting excessively rapid release. Therefore, the obtained release profile most likely results from the combined effects of polymer network organization, swelling behavior, and physical interactions between TAX and the hydrogel matrix. Moreover, the sustained release observed after prolonged incubation may be beneficial for implant surface functionalization, as the gradual release of bioactive compounds could support longer-lasting biological activity at the implantation site. This controlled-release profile may also explain why TAX-βCD-modified hydrogels maintained favorable cytocompatibility, as gradual TAX release reduces the risk of local concentration-related cytotoxicity while preserving its potential antioxidant and osteomodulatory activity.

The indirect cytotoxicity studies demonstrated that CLG and TAX-βCD-modified hydrogels exhibited improved cytocompatibility toward MC3T3-E1 preosteoblasts compared with unmodified systems. The enhanced biological response may be associated with the presence of CLG, which supports cell–material interactions, and TAX, known for its antioxidant and anti-inflammatory properties. Cell viability values exceeding 100% should be interpreted with caution. Although they may suggest a favorable cellular response, they cannot be unequivocally attributed to enhanced metabolic activity or proliferation. This is particularly important for TAX-βCD-containing systems, because TAX is a flavonoid with antioxidant and redox-active properties. Such compounds may potentially interfere with XTT and resazurin assays through direct or indirect reduction in assay reagents or by affecting the redox balance of the culture environment. Importantly, βCD complexation improves TAX stability and modulates its release, but it does not remove the intrinsic redox character of TAX. Therefore, in the present study, values above 100% are considered primarily evidence of cytocompatibility and the absence of cytotoxicity according to the ISO 10993-5 criteria. Additional acellular controls containing TAX or TAX-βCD extracts without cells, as well as complementary methods such as DNA quantification, direct cell counting, or live/dead staining, would be required to confirm whether these values reflect true stimulation of cellular metabolism or proliferation. Combined with the observed hydrophilicity and structural stability, these findings suggest that the developed coatings may provide a biologically favorable environment for potential orthopedic and dental applications.

Overall, the results presented provide a solid basis for further biological evaluation and optimization of multifunctional hydrogel coatings intended for biomedical applications. The obtained physicochemical and biological results demonstrate the high potential of the developed multifunctional coatings for orthopedic and dental applications. At the same time, further studies involving advanced crystallographic analyses and extended biological evaluation may provide additional insight into the structural and biological behavior of the developed systems. In particular, future investigations involving direct cell–material interactions could further support the applicability of the proposed coatings in biomedical engineering.

## 4. Materials and Methods

### 4.1. Materials for Synthesis of Hydroxyapatite

The synthesis of Ca_10−x_Mg_x_(PO_4_)_6_(OH)_2_ was carried out using calcium nitrate tetrahydrate (Ca(NO_3_)_2_·4H_2_O, Chempur, Piekary Śląskie, Poland), ammonium hydrogen phosphate ((NH_4_)_2_HPO_4_, Chempur, Piekary Śląskie, Poland), and magnesium nitrate hexahydrate (Mg(NO_3_)_2_·6H_2_O, Chempur, Piekary Śląskie, Poland). Ammonium hydroxide (NH_4_OH, Chempur, Piekary Śląskie, Poland) and double-distilled water were additionally used as auxiliary reagents. The composites were fabricated using the previously synthesized HAp, carboxymethylcellulose sodium salt (average molecular weight ~250,000; CAS: 9004-32-4), and a titanium alloy (Ti-6Al-4V, grade 5, Ti64).

### 4.2. Preparation of Magnesium-Substituted HAp Suspension

A modified co-precipitation approach was applied to synthesize magnesium-substituted HAp. The preparation of Ca_10−x_Mg_x_(PO_4_)_6_(OH)_2_ was conducted at room temperature, with the magnesium substitution level set at x = 0.1. The (Ca + Mg)/P molar ratio was maintained at the stoichiometric value of 1.67. An aqueous solution containing Ca(NO_3_)_2_·4H_2_O and Mg(NO_3_)_2_·6H_2_O was gradually introduced dropwise into a separate solution of (NH_4_)_2_HPO_4_ under continuous stirring. The pH of the reaction system was maintained at 11 by adding NH_4_OH. The mixture was then stirred at 80 °C for 12 h to ensure complete reaction. Following the reaction, the precipitate was collected by centrifugation and subsequently redispersed in deionized water for further processing. The resulting product was then dried in a laboratory oven, and the dried material was subsequently used in further experimental procedures.

### 4.3. Preparation of Ti/HAp-Mg Biomaterials

Carboxymethylcellulose (CMC) was employed as both a binder and a pore-forming agent. Two powder mixtures with varying contents of synthesized HAp were first prepared. The mixtures were homogenized using a PULVERISETTE 6 classic line planetary mill equipped with a zirconia bowl and balls, operating at 250 rpm in four cycles with 10 min intervals. Ti/HAp composites were then formed by cold uniaxial pressing of the powders into cylindrical samples (13 mm diameter, 4 mm height) under a load of 15 tons. Before sintering, the samples were dried at 300 °C for 2 h to remove organic components from CMC. Sintering was carried out at 1100 °C for 3 h under an argon atmosphere, with a heating and cooling rate of 2 °C/min, using a Nabertherm furnace (Nabertherm GmbH, Lilienthal, Germany).

### 4.4. Materials for Synthesis of Coatings 

The reagents used for the synthesis and characterization of the hydrogel materials are summarized in Table 4. All chemicals were used as received without further purification.

### 4.5. Preparation of Hydrogel Materials

Hydrogel materials were synthesized via UV-induced photopolymerization. In the preliminary stage, various polymer matrices (PVP, PEG, and PVP/PEG blends) were tested at concentrations of 5%, 10%, and 15% (*w*/*v*). The precursors were prepared by dissolving the polymers in deionized water using magnetic stirring. Subsequently, the crosslinking agent (0.6 mL or 1 mL of PEGDA 575/700), the photoinitiator (15 or 25 µL), and CLG were added. The solutions were poured into molds and cured under a UV lamp for 300 s. Based on visual assessment (homogeneity, structural integrity, and absence of cracks), three optimal compositions were selected for further studies (Table 5).

### 4.6. Functionalization with Bioactive Compounds

Two independent methods were employed to incorporate TAX into the hydrogel structures:

In situ incorporation of TAX-βCD complex: A TAX (TAX-βCD) inclusion complex was prepared in a 1:1 molar ratio. βCD was dissolved in deionized water (40–45 °C), and a TAX solution (50 mg in 1 mL ethanol) was added dropwise under continuous stirring in the dark. After ethanol evaporation, the solution was frozen at –80 °C and lyophilized for 47 h. The resulting powder was dissolved in water (20 mg/mL) and added to the hydrogel precursor (1.3 MIX composition) before UV crosslinking.

Post-loading method: Pre-crosslinked hydrogel discs were immersed in a TAX solution (20 mg/mL in PBS with 1% *v*/*v* ethanol). Samples were incubated in 0.25, 0.5, or 1.0 mL volumes at 25 °C for 60 min in the dark. After loading, samples were rinsed with PBS and dried at 25 °C for 60 min.

### 4.7. Substrate Preparation and Coating Techniques

Two types of titanium-based substrates were used: porous Ti-HAp composites and solid Ti6Al4V sheets. Substrates were mechanically polished (180- and 360-grit paper), cleaned in acetone and deionized water, and treated in an ultrasonic bath, then dried at 100 °C.

To improve adhesion, all substrates were pre-treated with a polydopamine (PDA) layer. Substrates were immersed in a dopamine solution (2 mg/mL in 10 mM Tris buffer, pH 8.5) for 30 min at room temperature, then rinsed with deionized water and air-dried.

**Figure 26:**
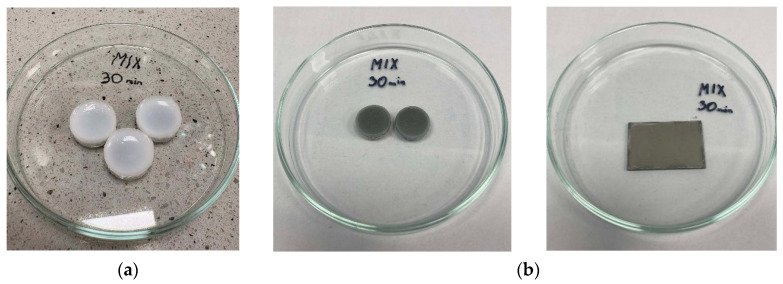
Hydrogel coatings obtained using different deposition techniques: (**a**) dip-coating; (**b**) controlled drop-casting, yielding thin coating layers applied onto metallic substrates, including porous and solid materials.

The hydrogel coatings were applied using two techniques:

- Dip-coating: Substrates were immersed in the hydrogel precursor and subsequently cured under UV light (Figure 26a).
- Controlled drop-casting: A specified volume of the precursor was dispensed onto the substrate using an automatic pipette, spread to ensure a uniform layer, and immediately crosslinked under UV radiation (Figure 26b).

### 4.8. Incubation Studies in Physiological Fluids

Incubation tests were performed to evaluate interactions between the hydrogel matrices and fluids that mimic the human physiological environment. The study utilized SBF (pH 7.64; Table 6), PBS (pH 7.45; Table 7), and AS (pH 6.04; Table 8). Samples were placed in sterile containers with 50 mL of each fluid and incubated at 37 °C for 14 days. The pH and electrical conductivity values were monitored at regular intervals using a CX-705 ELMETRON multifunctional meter.

### 4.9. Swelling and Degradation Analysis

The sorption capacity of the hydrogels was evaluated using the swelling ratio (α). Pre-weighed dry samples (m0) were immersed in SBF, PBS, and AS at 37 °C. At specified time intervals (15 min, 30 min, 1 h, 24 h, 4 days, and 14 days), samples were removed, blotted with filter paper to remove excess surface liquid, and weighed (mt). The swelling degree was calculated according to Equation (1):

(1) $$\propto =\frac{m_{t}-m_{0}}{m_{0}}$$

where *m_t_* is the mass of the swollen hydrogel, and *m*_0_ is the initial dry mass.

### 4.10. Scanning Electron Microscopy

The surface morphology, topography, and structural integrity of the developed hydrogel coatings were examined using a JSM-IT200 scanning electron microscope (JEOL, Tokyo, Japan). The analysis was performed in high-vacuum mode with an accelerating voltage of 15.0 kV. Before imaging, the samples were mounted on aluminum stubs with conductive carbon tape and coated with a 10 nm-thick layer of gold using a sputter coater to ensure electrical conductivity and minimize thermal damage to the polymer matrix.

Before SEM analysis, the hydrogel samples were dried under ambient conditions to remove residual moisture. For samples incubated in SBF, specimens were gently rinsed with distilled water to remove loosely bound salts and then dried before analysis. The SEM observations were therefore performed on dried hydrogel materials and should be interpreted mainly as qualitative morphological characterization.

### 4.11. Fourier Transform Infrared Spectroscopy

The chemical structure and functional group presence in the raw materials (PVP, PEG, TAX, CLG) and the crosslinked hydrogels were analyzed using a Nicolet iS5 FTIR spectrometer (Thermo Scientific, Waltham, MA, USA). The device was equipped with an attenuated total reflectance (ATR) accessory featuring a high-pressure monolithic diamond crystal (ID7). Spectra were recorded over the wavenumber range 4000–400 cm^−1^ at a resolution of 4 cm^−1^, with 32 scans averaged per spectrum. Background measurements were performed before each sample analysis, and the diamond crystal was thoroughly cleaned with high-purity acetone between measurements to prevent cross-contamination.

### 4.12. X-Ray Diffraction

The phase composition of the synthesized HAp was analyzed using X-ray diffraction (XRD). The measurements were carried out with a Malvern Panalytical Aeris diffractometer (Malvern PANalytical, Almelo, The Netherlands) equipped with a PIXcel1D-Medipix3 detector and a Cu Kα radiation source. Diffraction patterns were recorded in the 2θ range of 10–100° with a step size of 0.0027166° and an acquisition time of 340.425 s per step to ensure high-resolution diffraction data.

The degree of crystallinity of the synthesized HAp was determined based on XRD data using the crystallinity coefficient (X_c_), calculated as the ratio of the integrated area of crystalline peaks to the total area corresponding to both crystalline and amorphous phases, according to the following Equation (2):

(2) $$X_{c}=\frac{A_{c}}{{(A}_{c}+A_{a})}\cdot 100\%$$

where X_c is_ the degree of crystallinity (%); A_c_ is the area under crystalline peaks; and A_a_ is the area under amorphous peaks.

The crystallite size (average size of crystallite areas in the sample) was calculated using Scherrer’s Equation (3):

(3) $$B_{k}=\frac{K\lambda }{D_{hkl}cos\theta }$$

where *B_k_* is the reflectance width, dependent on crystallite size, [rad]; *K* is a constant associated with the shape, [-]; *λ* is the radiation wavelength, [Å]; *D* is the size of crystallites in the direction perpendicular to the (hkl), [Å]; and *θ* is Bragg’s angle [°].

### 4.13. UV–Vis Spectroscopy

The release profiles of TAX from the functionalized coatings and the TAX-βCD complex stability were evaluated using a GENESYS 180 UV–Vis spectrophotometer (Thermo Scientific). To immobilize the active substance within the polymer structure, a stock solution of TAX was prepared by dissolving 15 mg of the substance in 6 mL of an appropriate solvent, achieving a homogeneous concentration of 2.5 mg/mL. Following complete homogenization, the solution was micro-dispensed into standardized casting molds in precise 1 mL aliquots, where the polymer matrix was subsequently cross-linked. This technological procedure ensured that each synthesized matrix was impregnated with a constant, strictly defined, and identical initial payload of TAX amounting to exactly 2.5 mg. For the long-term in vitro release kinetic studies, three independent polymer matrices were selected, ensuring a triplicate experimental design (n = 3). The entire, intact matrix possessing a precisely determined dry mass of 0.2299 g (corresponding to an actual matrix loading of 10.87 mg of TAX per 1 g of dry polymer) was placed in its entirety into dissolution vessels containing 30 mL of PBS. The PBS medium served as the receptor phase to mimic physiological conditions (T = 37 °C). At predefined time intervals (0, 15, 30, 45 min and 1, 2, 3, 4, 5, 24, and 48 h), sample aliquots were withdrawn. The concentration of the released TAX was quantitatively determined via UV–Vis spectrophotometry at a wavelength of λ = 290 nm, corresponding to the absorption maximum of the studied polyphenol. The concentration of the released TAX was calculated using the previously established calibration curve equation (A = 56.158 c − 0.052, R^2^ = 0.98), and the final data were expressed as the cumulative release (%) over time.

### 4.14. Dynamic Mechanical Analysis

The mechanical properties of the hydrogel coatings were evaluated using a Discovery DMA 850 Dynamic Mechanical Thermal Analyzer (TA Instruments, New Castle, DE, USA). This system was employed to determine the material’s response to controlled dynamic loading. Prior to the measurement, the precise physical dimensions Z (height and width) of each hydrogel specimen were measured and recorded to ensure accurate stress/strain calculations. The analysis was conducted under an initial preload of 0.01 N to ensure proper contact between the instrument’s geometry and the sample surface. All measurements were performed at a stabilized ambient temperature of 24 °C.

### 4.15. Biological Tests

#### 4.15.1. Cell Culture Condition

For the in vitro studies, the murine preosteoblastic cell line MC3T3-E1 Subclone 14 (ATCC-LGC Standards, Teddington, UK; CRL-2594) was employed. Cells were maintained in α-Minimum Essential Medium (α-MEM) enriched with 10% fetal bovine serum (FBS). The culture medium was additionally supplemented with penicillin (100 U/mL), streptomycin (100 μg/mL), l-glutamine (2 mM), and amphotericin B (1.25 μg/L). Cell cultures were kept under standard incubation conditions at 37 °C in a humidified atmosphere containing 5% CO_2_ to ensure proper growth and viability. Cells were routinely passaged every 2–3 days once they reached approximately 85–90% confluency.

#### 4.15.2. Indirect Cytotoxicity Test

The indirect cytotoxicity studies were conducted in accordance with the ISO 10993-5 recommendations, using extracts derived from hydrogel composite materials. Extract preparation followed the ISO 10993-12 [63] protocol, in which 100 mg of each biomaterial sample was immersed in 1 mL of medium and incubated for 24 h at 37 °C in a humidified atmosphere containing 5% CO_2_. At the same time, MC3T3-E1 cells were seeded in 96-well culture plates at a concentration of 2 × 10^4^ cells per well and cultured in 200 µL of α-MEM for 24 h under identical incubation conditions. After this preincubation stage, the culture medium was aspirated and substituted with 200 µL of the previously prepared biomaterial extracts. The cells were subsequently incubated for another 24 h at 37 °C in a humidified 5% CO_2_ atmosphere. To establish the positive control, selected wells were treated with 0.1% (*v*/*v*) Triton X-100 for 15 min, followed by two washes with DPBS. Cells maintained exclusively in α-MEM served as negative controls. Cell viability was subsequently evaluated using the XTT colorimetric assay (XTT tetrazolium salt assay) and the resazurin-based NRU test, as outlined in the following section.

#### 4.15.3. XTT Assay

Initially, the XTT reagent solution was freshly prepared by dissolving XTT in sterile Hanks’ balanced salt solution to obtain a final concentration of 1 mg/mL. Next, PMS solution (5 mM stock) was introduced at a volume of 5 μL per mL of reagent. Following the removal of the culture medium, 100 μL of the prepared XTT mixture was dispensed into each well of the 96-well plate. The plate was then incubated for 4 h at 37 °C. After incubation, absorbance values were recorded at 450 nm, with 630 nm as the reference wavelength, using a BioTek 800 TS microplate spectrophotometer (Agilent, Winooski, VT, USA). All measurements were performed in at least triplicate. Cell viability was subsequently calculated according to Equation (4):

(4) $${\%}_{viability,XTT}=\frac{{{(A}_{450}-A_{630})}_{tested\;cells}}{{{(A}_{450}-A_{630})}_{control\;cells}}$$

#### 4.15.4. Resazurin Reduction-Based Test

Following the incubation period, the extracts were aspirated, and the wells were gently rinsed with PBS. Subsequently, 200 μL of resazurin solution at a concentration of 100 µM was added to each well. The plates were then incubated for an additional 4 h in darkness at 37 °C in a humidified atmosphere containing 5% CO_2_. After incubation, absorbance readings were collected at wavelengths of 570 nm and 630 nm using a BioTek 800 TS microplate reader (Agilent, Winooski, VT, USA). Cell viability was expressed relative to the negative control, consisting of cells cultured on standard polystyrene plates in pure α-MEM medium. Each experiment was carried out at least three times. The percentage of viable cells was calculated according to Equation (5):

(5) $${\%}_{viability,resazurin}=\frac{{{(A}_{570}-A_{630})}_{tested\;cells}}{{{(A}_{570}-A_{630})}_{control\;cells}}$$

### 4.16. Statistical Analysis

Statistical analysis of the obtained results was carried out using one-way analysis of variance (ANOVA) to determine significant differences between the examined groups. Differences were regarded as statistically significant when the probability values reached * *p* < 0.05, ** *p* < 0.01, or *** *p* < 0.001. All calculations were performed using OriginPro 2019 software (OriginLab, Northampton, MA, USA).

## 5. Conclusions

This study demonstrates that PVP/PEG-based hydrogel coatings functionalized with CLG and TAX-βCD inclusion complex can be applied to modify titanium-based substrates with various surface characteristics, including both relatively smooth titanium sheets and porous composite surfaces (Ti6Al4V/Hap). The proposed coating strategy allowed for the formation of stable hydrogel layers and introduced bioactive functionality without compromising the overall physicochemical stability of the systems.

The obtained results indicate that magnesium modification influenced the structure of HAp, leading to reduced crystallinity and structural disorder typical of ion-modified apatites. Although complete Mg^2+^ substitution in the apatite lattice cannot be unequivocally confirmed, the developed Ti6Al4V/HAp-Mg composite showed surface features relevant for biomedical applications, including a hydrophilic character with an average contact angle of 54.15°, which is advantageous in terms of cell adhesion and potential osseointegration. The hydrogel coatings remained stable during incubation in a simulated physiological environment, without significant destabilization of pH, conductivity or mass. At the same time, the TAX-βCD-containing systems provided the gradual release of taxifolin, confirming that the coatings can serve not only as passive surface layers, but also as local carriers of bioactive flavonoid compounds. Biological evaluation using MC3T3-E1 preosteoblasts showed that the developed systems were not cytotoxic and that the incorporation of CLG and TAX-βCD was associated with a favorable preliminary cellular response. These findings suggest that the combination of PVP/PEG hydrogel matrix, CLG and complexed taxifolin is a promising direction for functionalization of titanium-based implant materials.

Overall, the developed coatings represent a versatile platform for further study of bioactive implant surfaces. Future work should focus on direct cell–material interactions, osteogenic differentiation, antibacterial activity, sustained release and in vivo performance to better define their potential in bone-related applications.

## Figures and Tables

**Table 1:** Crystallinity parameters of HAp powder.

Parameter	Hydroxyapatite
Crystallite size (nm)	9.80
Degree of crystallinity (%)	69.54

**Table 2:** Contact angle measurements of Ti6Al4V/HAp composites modified with Mg, including left, right, and average values with representative images of the analyzed droplets.

Sample	Left Contact Angle (°)	Right Contact Angle (°)	Average Contact Angle (°)	Contact Angle Image
Ti6Al4V-5% HAp-Mg-5% CMC	52.19	56.11	54.15	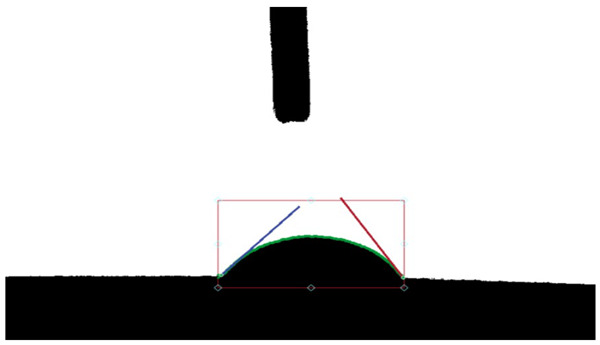

**Table 3:** Young’s modulus values for selected hydrogel materials.

Sample Designation	Young’s Modulus (kPa)
PEG	729 ± 26
PVP	1379 ± 178
MIX	1371 ± 205
MIX + CLG	2530 ± 136

**Table 4:** List of reagents, manufacturers, and specifications.

Reagent	Manufacturer	Specification
Polyethylene glycol (PEG)	Acros Organics	Mw ≈ 8000 g/mol, water-soluble
Poly(vinylpyrrolidone) (PVP)	Acros Organics	Mw ≈ 8000 g/mol, water-soluble
Polyethylene glycol diacrylate 575 (PEGDA 575)	Sigma Aldrich	Crosslinking agent, Mn = 575 g/mol, ρ = 1.12 g/mL
Polyethylene glycol diacrylate 700 (PEGDA 700)	Sigma Aldrich	Crosslinking agent, Mn = 700 g/mol, ρ = 1.12 g/mL
2-Hydroxy-2-methylpropiophenone	Sigma Aldrich	Photoinitiator, 97%, ρ = 1.077 g/mL
(+)-Taxifolin (Dihydroquercetin)	TCI	Mw = 304.25 g/mol, purity ≥ 95%, powder
β-Cyclodextrin	AmBeed	Mw ≈ 1135 g/mol, purity ≥ 98%, powder
Collagen (Bovine Achilles tendon)	Sigma Aldrich	Type I, powder
Minimum Essential Medium Eagle, Alpha Modification, α-MEM	Sigma Aldrich	with sodium bicarbonate and Earle’s salts; without l-glutamine, ribonucleosides, and deoxyribonucleosides
Fetal Bovine Serum (FBS)	Sigma Aldrich	
Penicillin-Streptomycin	Sigma Aldrich	10,000 units penicillin and 10 mg streptomycin/mL; 0.1 μm filtered; BioReagent
Amphotericin B	Sigma Aldrich	250 μg/mL in deionised water; 0.1 μm filtered; BioReagent
l-glutamine	Sigma Aldrich	200 mM; BIOXTRA
Trypsin-EDTA	Sigma Aldrich	0.25% Trypsin in 1 mM EDTA in HBSS
Dulbecco’s Phosphate-Buffered Saline (DPBS)	Sigma Aldrich	Tablets
Hanks’ Balanced Salt Solution (HBSS)	Sigma Aldrich	modified
XTT Sodium Salt	Sigma Aldrich	BioReagent; suitable for cell culture
Phenazine Methosulfate	Sigma Aldrich	-
Resazurin Sodium Salt	Sigma Aldrich	-
Trypan Blue	Sigma Aldrich	-
Formaldehyde	Sigma Aldrich	36.5–38.0% in H_2_O; for molecular biology
Triton X-100	Sigma Aldrich	-
Ethanol	Sigma Aldrich	96%
Hydroxyapatite	Sigma Aldrich	Reference material

**Table 5:** Composition of the selected hydrogel matrices (volume of components per sample).

Sample ID	Polymer Solution (5% *w*/*v*)	Collagen (g)	PEGDA 575	Photoinitiator
1.1 PVP	3.0 mL PVP	-	0.6 mL	15 µL
1.2 PEG	3.0 mL PEG	-	0.6 mL	15 µL
1.3 MIX	1.5 mL PVP + 1.5 mL PEG	-	0.6 mL	15 µL
2.1 PVP + col	3.0 mL PVP	0.0117	0.6 mL	15 µL
2.2 PEG + col	3.0 mL PEG	0.0117	0.6 mL	15 µL
2.3 MIX + col	1.5 mL PVP + 1.5 mL PEG	0.0117	0.6 mL	15 µL

**Table 6:** Chemical composition of simulated body fluid (SBF) at pH 7.64.

Order	Components	Amount (g/L)
1	NaCl	8.035
2	NaHCO_3_	0.355
3	KCl	0.225
4	K_2_HPO_4_ · 3H_2_O	0.1763
5	MgCl_2_ · 6H_2_O	0.311
6	HCl 1 M	40 mL
7	CaCl_2_	0.292
8	Na_2_SO_4_	0.072
9	C_4_H_11_NO_3_	6.118
10	HCl 1 M	0–5 mL (for pH adjustment)

**Table 7:** Chemical composition of phosphate-buffered saline (PBS) at pH 7.45.

Order	Component	Amount (g/L)
1	NaCl	8.000
2	KCl	0.200
3	Na_2_HPO_4_	1.150
4	KH_2_PO_4_	0.200

**Table 8:** Chemical composition of artificial saliva (AS) at pH 6.04.

Order	Component	Amount (g/L)
1	NaCl	0.4000
2	KCl	0.4000
3	CaCl_2_ · 2H_2_O	0.6841
4	Na_2_HPO_4_ · H_2_O	0.6780
5	Na_2_S · 9H_2_O	0.0050
6	Urea	1.0000

## Data Availability

Data that support the findings of this study are contained within the article.
